# Fatigue Crack Monitoring Method Based on the Lamb Wave Damage Index

**DOI:** 10.3390/ma17153836

**Published:** 2024-08-02

**Authors:** Muyang He, Chengwu Dong, Xiaodan Sun, Jiayi He

**Affiliations:** 1 School of Civil Engineering, Harbin Institute of Technology, Harbin 150090, China; 23s033070@stu.hit.edu.cn; 2 College of Aerospace and Civil Engineering, Harbin Engineering University, Harbin 150001, China; 15776456378@163.com; 3 School of Information Science and Engineering, Harbin Institute of Technology (Weihai), Weihai 264209, China; 23s030127@stu.hit.edu.cn

**Keywords:** Lamb wave, fatigue crack, damage index, wavefield propagation, thick plates, structural health monitoring (SHM)

## Abstract

For practical engineering structures, fatigue is one of the main factors affecting their safety and durability. Under long-term service conditions, the minor damage will be affected by fatigue loading and expand to macroscopic cracks, affecting the structure’s service performance. Based on the sensitivity of Lamb waves to minor and initial damage, a damage monitoring method for fatigue crack propagation is proposed. By carrying out fatigue crack propagation tests under constant amplitude loading, the Paris equation of 316L steel and damage signals at different crack growth stages were obtained. Combined with damage monitoring tests and finite element analysis, the relationship between the phase damage index (PDI), amplitude damage index (ADI), signal correlation coefficient, and fatigue crack propagation length was studied. Compared with PDI and ADI, the signal correlation coefficient is more sensitive to crack initiation, which can be selected as the damage monitoring index in the initial stage of crack growth. With the increase of fatigue crack propagation length, the peak time of the direct wave signal gradually moves backward, which shows an obvious phase change. In the whole fatigue crack growth stage, PDI and crack length show a monotonically changing trend. By using the stress intensity factor as the conversion parameter, a prediction model of the fatigue crack propagation rate based on PDI was established. Compared to the fatigue crack propagation rate measured by experiments, the relative error of the predicted results is 10%, which verifies the accuracy of the proposed damage monitoring method.

## 1. Introduction

Fatigue, one of the primary factors leading to the failure of engineering structures, has gained significant research attention. Fatigue failure is mainly due to the gradual propagation of very small cracks generated in the material during repetitive loading processes, which eventually leads to structural failure [1]. Structural health monitoring (SHM), fatigue damage analysis, and life prediction technologies are important innovations in the field of modern engineering. These technologies can detect the damage early and take maintenance measures to ensure the safety of the structure during its service [2,3]. In addition, monitoring data and existing theoretical studies can be used to predict damage development trends and even forecast the remaining life of the structure. Lamb waves are widely used in acoustic emission monitoring methods based on their propagation mechanism in waveguides, where guided waves are excited in the structure by an exciter and received by sensors at different positions on the structure [4]. For engineering structures with certain damage, the received signals reflect changes in the amplitude and mode of the guided waves. These changes can be used to locate and image damage [5,6]. Furthermore, by extracting features from these received signals, damage can be quantitatively assessed [7,8].

Giridhara et al. [9] developed a damage detection and imaging method based on the symmetry and signal patterns of Lamb waves using a sensor path in the neighborhood, enabling fast localization and quantification of damage severity. Haider et al. [10] discussed the interaction of Lamb waves with cracks in reinforced bars. They developed a global–local analytical method to predict the scattering behavior of Lamb waves in complex structures, confirming its accurate crack detection capability. Tua et al. [11] proposed a comprehensive method for locating and determining the linear crack range in homogeneous plates based on time-of-flight analysis of Lamb wave propagation. Biemans et al. [12] effectively identified fatigue cracks of 6 to 7 mm using wavelet transform to extract feature signals. Loutridis et al. [13] introduced a dual crack identification method for beam cracks based on wavelet analysis, analyzing the fundamental vibration modes of dual crack cantilever beams using continuous wavelet transform to estimate the crack position and depth. Douka et al. [14] estimated the position and size of beam structure cracks using wavelet analysis and defined a strength factor to predict crack size. Lu et al. [15] conducted finite element analysis and experimental studies to investigate the interaction between Lamb waves of different frequencies and cracks in aluminum plates, which facilitated the precise evaluation of through-thickness cracks. Additionally, Gangadharan et al. [16] quantitatively evaluated damage size and material degradation parameters using the wavelet transform and the Hilbert–Huang transform. He et al. [17] found a correlation between fatigue crack length and three main features of the signal: the coefficient of correlation, amplitude variation, and phase variation. Wang et al. [18] studied a model evaluation method using Lamb waves to predict structural fatigue life, conducted Lamb wave face tests during model development, and extracted three damage-sensitive features—the normalized energy, phase change, and correlation coefficient—from Lamb wave data for quantifying crack size. Xu et al. [19] investigated the acoustic emission signal characteristics of fatigue crack initiation and propagation in three types of rail materials under different fatigue load conditions. They proposed a method to determine the fatigue crack propagation rate.

Currently, there is a lack of research on the monitoring of cracks in thick plates using Lamb waves. Greve et al. [20] discussed waves created in relatively thick plates by edge excitation, examined the transfer of energy from leading to trailing pulses, and studied the interaction of these trailing pulses with cracks. Sun et al. [21] presented a diagnosis procedure on thick steel beams with a thickness of 34 mm, and discussed the frequency and the number of cycles of the diagnostic waveform. Grisso et al. [22] tested the feasibility of monitoring the structural integrity of welded thick aluminum plates by using the SHM methods: impedance and Lamb wave analyses. Schaal et al. [23] developed an analytical framework to analyze the scattering of an incident ultrasonic Rayleigh wave at a delamination-like discontinuity located near the surface of a thick plate. Shao et al. [24] proved that trailing pulses are a powerful tool for the damage detection of thick plates, and the blind pulse compression method for trailing pulses does not need material properties.

This study explored the relationship between the fatigue crack growth model in thick plates and Lamb wave damage indices, revealing consistent patterns. A crack growth monitoring model that links phase damage indices to stress intensity factor ranges was established. This model can determine crack propagation rates through phase damage indices, providing valuable guidance for Lamb wave monitoring of fatigue crack propagation. The paper is organized as follows: Section 2 introduces the derivation of the phase damage index. Section 3 describes the experimental setup, the excitation signals employed in the experiments, and the relationship between the phase damage index and the Paris equation. This relationship is then leveraged to predict the rate of crack propagation using the damage index. Section 4 presents our numerical simulation analyses, examining the interaction between Lamb waves and fatigue cracks, thereby verifying the effectiveness of the damage index in monitoring fatigue crack propagation.

## 2. Damage Index

When crack propagation occurs in a specimen, Lamb waves undergo scattering and diffraction. Directly estimating crack size using measured signals is challenging due to various influencing factors, such as environmental noise, equipment precision, and material properties. Therefore, in practical applications, it is often necessary to perform a series of denoising processes, and window function truncations need to be performed on these measurement data to obtain the changing characteristics of the Lamb wave signal at the receiving end, and for estimating the stage of crack propagation. This study investigated changes in the correlation coefficients of receiving-end Lamb waves, variations in amplitude, and phase changes. The correlation coefficient depends solely on changes in signal shape [25]. These changes are due to crack propagation, which directly affects the shape of the Lamb wave signal at the receiving end, leading to variations in the correlation coefficient between it and the unmodified signal. When the structure is healthy, the signal remains unaffected, and the correlation coefficient is 1. However, with an increase in damage size, the correlation coefficient will change accordingly. This coefficient serves as an indicator of the presence of damage in the component, and this study also examines its relationship with the extent of damage. The following formula is used to calculate the coefficient, *I_C_*:

(1) $$I_{C}(X_{h},X_{d})=\frac{\mathrm{Cov}(X_{h},X_{d})}{\sigma_{X_{h}}\sigma_{X_{d}}}$$

where $X_{h}$ is the Lamb wave signal without damage, $X_{d}$ is the Lamb wave signal with a crack, $\mathrm{Cov}(X_{h},X_{d})$ is the covariance between health and damage signals, $\sigma_{X_{h}}$ is the standard deviation of the signal without damage, and $\sigma_{X_{d}}$ is the standard deviation of the signal with a crack.

**Figure 1:**
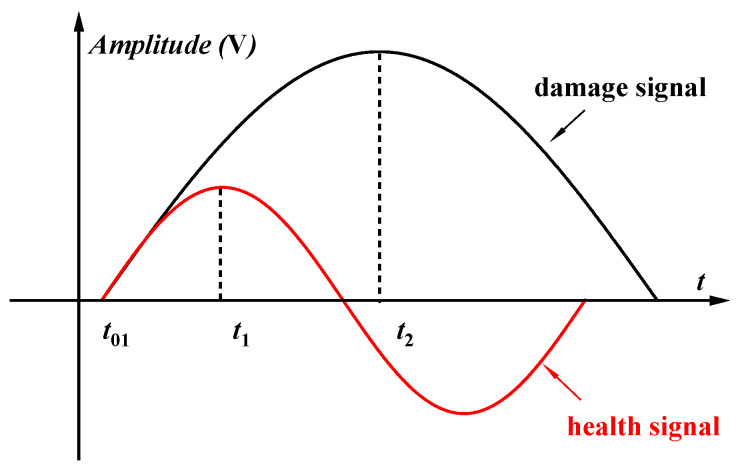
Fatigue cracks were monitored (using the lamb wave damage index) to obtain the Lamb wave damage index.

During the crack propagation process in CT specimens, Lamb wave signals of different crack lengths were extracted to quantitatively characterize the crack damage using the amplitude damage index (ADI) and phase damage index (PDI) [26]. The amplitude reflects the energy carried by the signal, which changes with increasing crack length, thus altering the amplitude damage index. The phase represents the change in flight time, which increases with crack propagation, leading to an increase in the phase damage index. This study primarily focuses on the mid-stage of crack extension, specifically the damage indices in the medium-rate period of crack propagation, denoted as *I_A_* for ADI and *I_P_* for PDI.

(2) $$I_{A}=ADI=\frac{A_{d}}{A_{h}}$$

(3) $$I_{P}=PDI=P_{d}-P_{h}$$

where *A_h_* is the Lamb wave signal amplitude without damage, and *A_d_* is the Lamb wave signal amplitude with a crack. As shown in Figure 1, the phase point of π/2 is chosen as the reference point. *t*_1_ can be obtained from the signal, and the phase value of the healthy signal $P_{h}$ is π/2. Therefore, *t*_01_ is the zero-phase moment of the healthy signal and can be derived based on the formula $P_{h}=2\pi f(t_{1}-t_{01})$. Similarly, *t*_2_ is the arrival time of the π/2 point in the damage signal and can also be obtained from the signal, allowing us to calculate the phase value of the damage signal $P_{d}=2\pi f(t_{2}-t_{01})$. Consequently, the phase difference is $\Delta \theta =I_{P}=P_{d}-P_{h}$. The healthy signal means the signal collected from the undamaged plate, and the damage signal means the signal collected from the damaged plate.

## 3. Experiments

### 3.1. Fatigue Crack Propagation Experiment

**Figure 2:**
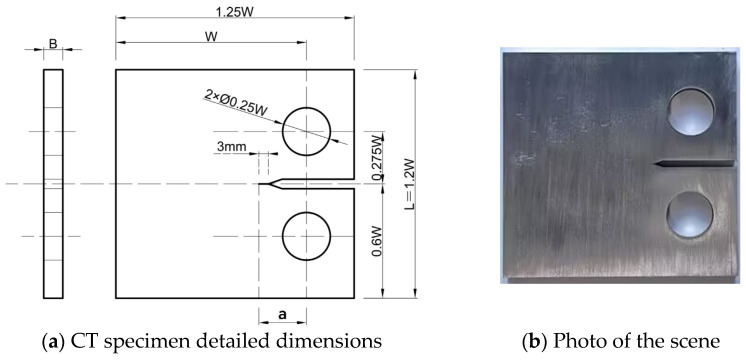
CT specimen detailed dimensions and photo of the scene.

The test specimens were designed according to the standard GB/T 6398-2017 [27] using compact tension (CT) specimens. Crack propagation tests were conducted on CT specimens made of 316L steel, and piezoelectric ceramic patches were attached to the CT specimens to monitor Lamb waves in real-time, as shown in Figure 2. The test material in this study was 316L steel, and the material parameters are shown in Table 1.

To facilitate the preformation of qualified fatigue cracks and ensure that the crack propagation path was a Type I crack, an artificial crack with a length of 3 mm was introduced at the top of the notch, which also served to prevent crack branching and its impact on the fatigue crack propagation rate. The crack was generated by manually operating the fatigue testing machine through fatigue loading. The applied load range in the test was 27 kN with a stress ratio of 0.1. The dimensions of the specimen at different positions were measured and averaged, as shown in Table 2. a represents the length of the pre-crack, which includes a notch and an artificial crack with a length of 3 mm.

**Figure 3:**
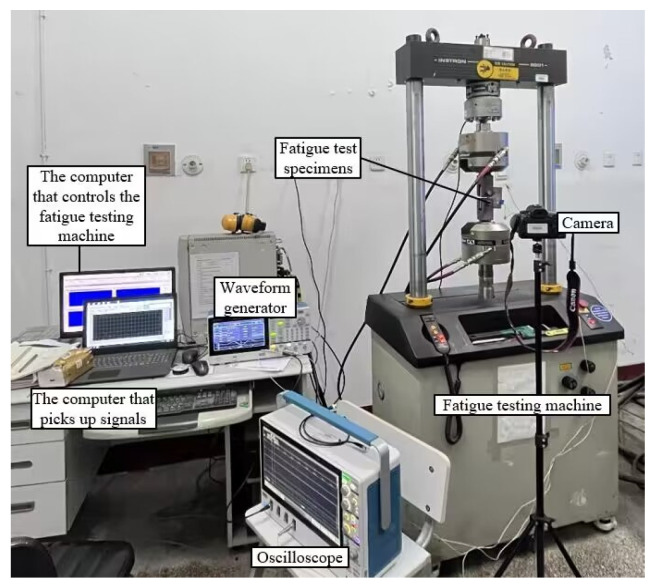
Online monitoring test platform for fatigue crack extension.

The testing platform consists of the following instruments: (1) an electrohydraulic servo fatigue testing machine (model INSTRON-8801, INSTRON Cooperation, Boston, MA, USA) with a loading frequency range of 0 to 100 Hz and a maximum load capacity of 100 kN, (2) a signal generator (model Tektronix AFG31052, Tektronix Cooperation, Beaverton, OR, USA), (3) a digital oscilloscope (model MOS545-BW-350, MOUSER ELECTRONICS, Shanghai, China) with four channels for waveform display, (4) piezoelectric ceramic patches with a diameter of 10 mm and thickness of 1 mm that are connected to the signal source (as the excitation end) and oscilloscope (as the receiving end) via shielded cables, with connections made through welding without exceeding the negative pole area of the piezoelectric ceramic patch, and (5) a camera for observing and recording crack propagation. Figure 3 shows the Online monitoring test platform for fatigue crack extension.

The test employs a loading frequency of 6 Hz, applying a sinusoidal cyclic fatigue load with a maximum load of 30 kN and a stress ratio of 0.1; hence, the minimum load is 3 kN. Every 100 cycles, the crack length is observed, a ruler is attached to the surface of the CT specimen for monitoring, and Lamb wave data are collected at crack extensions of 3 mm, 6 mm, 9 mm, 12 mm, 15 mm, and 18 mm at 3 mm intervals until the specimen reaches unstable failure. The collected Lamb wave signals are compared with the characteristics of Lamb wave signals without damage, and Lamb wave damage indices are calculated.

### 3.2. Excitation Signal

Narrowband excitation waves with narrow frequency bands and significantly lower bandwidths than the center frequency, effectively preventing wave packet overlap. Therefore, narrowband excitation signals are commonly used in finite element simulations and experiments. In this study, a Hanning window-modulated pulse narrowband sinusoidal wave signal is used, which is expressed as follows:

(4) $$y=0.5\times \left[1-\cos\left(\frac{2\times 2\pi \times f\times t}{n}\right)\right]\times \sin\left(2\times 2\pi \times f\times t\right)$$

where *n* represents the period of the excitation signal, and *f* represents the central frequency of the excitation signal, which is 90 kHz in this study.

**Figure 4:**
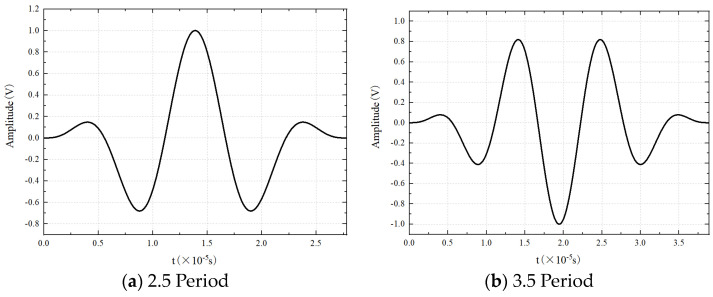
Sinusoidal excitation signal.

Setting an appropriate period in the excitation signal with distinct waveform features is crucial to enhance signal identification. Signals with period numbers of 2.5 and 3.5 exhibit clear waveform features, noticeable differences in peak numbers between the upper and lower sides, and larger amplitudes of the central peak compared to the smaller peaks on the left and right sides. Two types of cycles are initially tested, and 2.5 periods are ultimately chosen based on the experimental results. The excitation signals were applied directly to the specimen. The waveform is shown in Figure 4.

**Figure 5:**
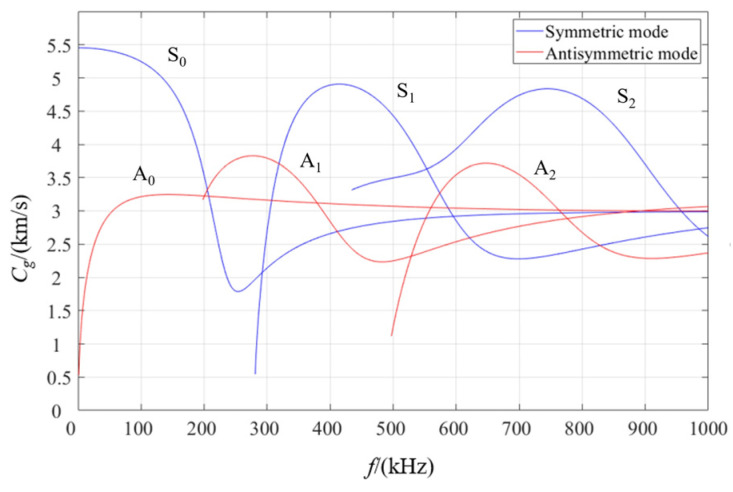
Group velocity dispersion curves of the 10 mm thick 316L steel plate.

Determining the excitation frequency based on the dispersion curve is a crucial step to ensure that only the required wave modes are excited. As shown in Figure 5, the cutoff frequency of the A_1_ mode for a 10 mm thick 316L steel plate is 180 kHz, while the cutoff frequency of the S_1_ mode is 240 kHz. Therefore, in order to ensure that only *A*_0_ and *S*_0_ modes of Lamb waves exist in the plate, the selected excitation frequency range should be limited to 0 to 180 kHz. This helps to avoid the generation of higher-order modes, simplifying signal analysis and focusing on the behavior of fundamental modes and corresponding data interpretation. In this section, the *A*_0_ mode and 90 kHz are used as examples for simulation. Due to the small size of the CT specimen, the direct wave easily overlaps with the waves reflected from the specimen boundary under faster group velocity propagation, which is not conducive to the study of damage signal characteristics. Therefore, antisymmetric excitation (*A*_0_ mode) with an excitation voltage of 10 V was selected as the Lamb wave excitation mode.

**Figure 6:**
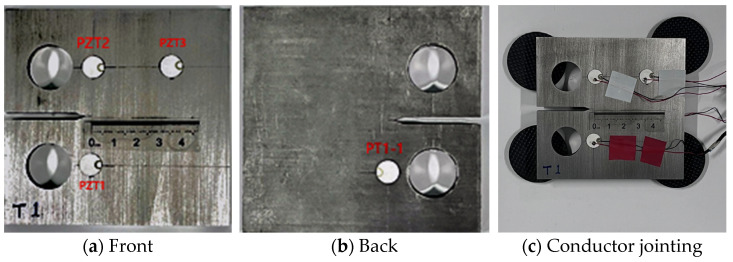
PZT arrangement on the CT specimen.

As shown in Figure 6, to investigate the influence of Lamb wave signals during fatigue crack propagation, piezoelectric ceramic transducers (PZTs) were arranged on both sides of the crack propagation path, the excitation end used an antisymmetric excitation mode with bilateral excitation, and PZT2 and PZT3 were set as receivers. PZT2 was mainly used to observe the signal characteristic changes of the direct wave passing through the crack, while PZT3 was mainly used to verify whether the Lamb wave propagation speed in the specimen was reasonable. The coordinates of PZT1 are (29 mm, 26 mm), PZT2 are (29 mm, 70 mm), and PZT3 are (70 mm, 70 mm). PZT was attached to the surface of the specimen using α-cyanoacrylate instant adhesive.

### 3.3. Crack Extension Model for the CT Specimen

The rate of fatigue crack propagation can be expressed in the Paris equation as follows:

(5) $$\frac{da}{dN}=C{\left(\Delta K\right)}^{m}$$

where $\frac{da}{dN}$ represents the rate of crack propagation, ΔK represents the range of the stress intensity factor, and *C* and *m* are fundamental parameters of material fatigue crack propagation performance [28].

According to the GB/T 6398-2017, the range of stress intensity factors for the CT specimens can be determined as follows:

(6) $$\Delta K=\frac{\Delta P}{B\sqrt{W}}\cdot \frac{\left(2+\alpha \right)}{{\left(1-\alpha \right)}^{3/2}}\left(0.886+4.64\alpha -13.32\alpha^{2}+14.72\alpha^{3}-5.6\alpha^{4}\right)$$

where α=a/W, and the application condition of Equation (6) is α≥0.2. In this paper, since the minimum value of a is 19 mm and B=80 mm, Equation (6) can be employed for the determination of ∆K. ∆K represents the stress intensity factor amplitude, Δ*P* is the load amplitude, and the dimensions of thickness *B* and width *W* are shown in Table 2.

**Figure 7:**
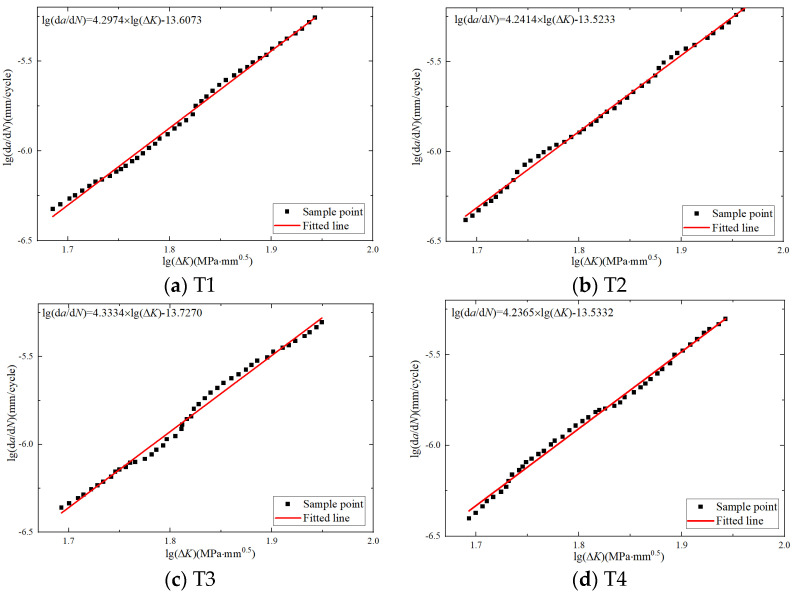
The relationship between lg(da/dN) and lg(ΔK) for each specimen.

A seven-point incremental polynomial method is employed for the local fitting of da/dN due to its high accuracy and wide applications. The relationship between lg(da/dN) and lg(ΔK) was plotted on a double logarithmic coordinate system. The linear fitting results are shown in Figure 7.

According to Figure 7, it was found that there is a linear relationship between the crack growth rate and the stress intensity factor range in the relationship between lg(da/dN) and lg(ΔK). By taking the base 10 logarithm on both sides of the Paris formula’s basic form, the following is derived:

(7) lg(da/dN)=lgC+mlgΔK

where the unit of lg(da/dN) is mm/cycle. After converting Equation (7) into the form of a linear function, i.e., y=px+q, the values of the parameters p and q can be determined through linear regression. Then, the material parameters *C* and *m* of the Paris equation can be deduced based on Equation (7), and the fitting results are presented in Table 3.

**Figure 8:**
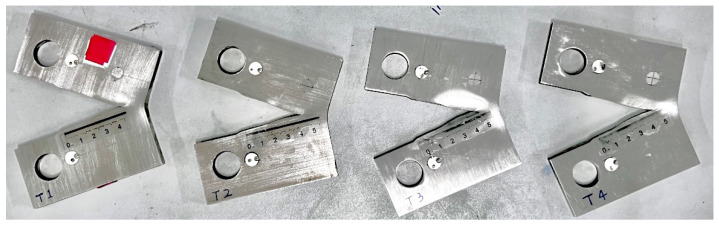
Specimens after the fatigue crack extension test (T1, T2, T3, and T4).

According to the parameters in the table, the differences between each parameter are very small. Therefore, the average values of the parameters and the coefficients of correlation for the four specimens are calculated, and the sample variance is shown in Table 4. During the experiment, when the fatigue cracking of the CT specimen extends to the later stage, rapid instability occurs due to the large range of loads and relatively low stress, which causes the fatigue testing machine to stop working. As a result, the CT specimen was not completely separated. The specimens after the test are shown in Figure 8.

### 3.4. Lamb Wave Signal Damage Index

**Figure 9:**
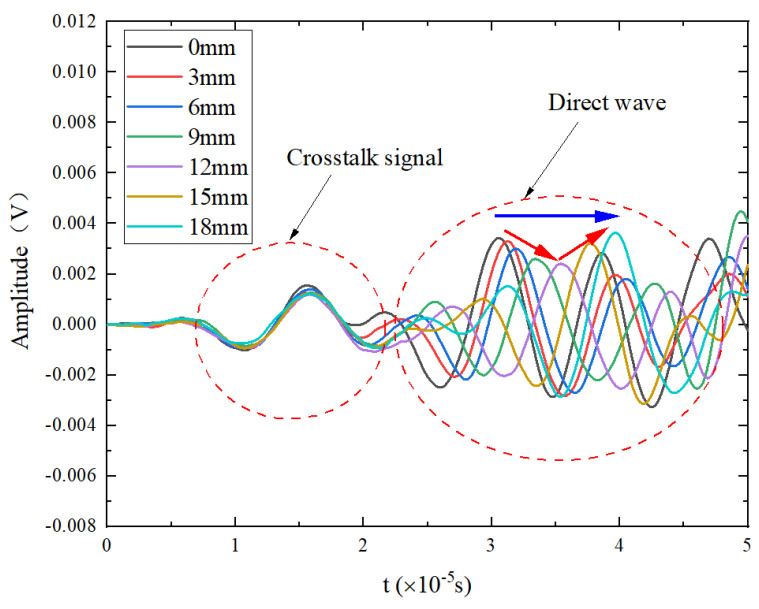
Monitoring signals of PZT1-PZT2.

Taking the Lamb wave signal received by PZT2 of specimen T3 as an example, Figure 9 shows the trend of Lamb wave direct wave signal variations with different crack lengths after filtering. Notably, the crack length here refers to the actual extended crack length, which is different from the nominal length mentioned earlier. The first waveform is the crosstalk signal synchronized with the excitation signal, and the second waveform is the direct wave signal. With an increase in cycle time, the crack length and width gradually increase, causing a delay in the peak time of the direct wave signal (blue arrow in Figure 9), which indicates a change in phase, i.e., PDI. The variation in the direct wave amplitude does not follow a monotonic pattern; the peak value decreases first and then increases, reaching a minimum value at a crack extension of 12 mm (red arrow in Figure 9).

**Figure 10:**
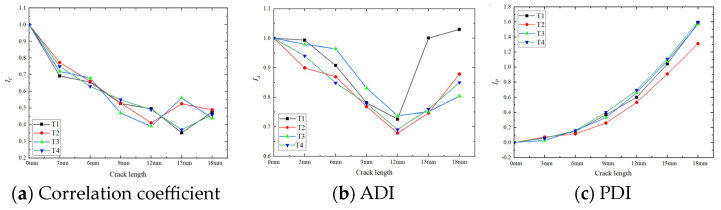
Variation in each index with crack length.

The Lamb wave signal correlation coefficient and damage index at the receiver end are calculated according to Equations (1)–(3). The relationship between each value and the crack length is shown in Figure 10. Figure 10a shows that the correlation coefficient decreases with crack extension, but this decrease is not strictly monotonic. During the extension from 0 mm to 12 mm, the correlation coefficients of the four specimens decrease with the increase in crack length. However, after 12 mm, they may increase or decrease, indicating that this index is not ideal for determining the stage of crack extension. When the coefficient changes from 0 mm to 3 mm, the decrease in the correlation coefficient from undamaged to damaged cracks is very significant, after which the trend becomes more gradual. Therefore, the correlation coefficient is highly sensitive to early crack stages and can be used as an indicator of the crack initiation stage.

Figure 10b shows that ADI first increases and then decreases with the increase in crack length. The minimum value is reached when the crack length is about 12 mm. Four specimens show the same pattern, with specimen T1 having greater amplitudes at 15 mm and 18 mm than the other three specimens. The change in amplitude is not significant as the crack length extends from 0 mm to 3 mm. After that, the change accelerates. During the stable crack propagation stage, the monotonicity of the amplitude damage index changes, indicating that the index is not sensitive to the early stage of crack initiation.

As shown in Figure 10c, the phase damage index increases monotonically with crack length, showing a gradual change from 0 mm to 9 mm, followed by a faster increase after 9 mm. The pattern is consistent across all four specimens, indicating that the phase damage index can be employed for monitoring crack development processes.

### 3.5. Analysis of the Causes of Trends in the Damage Index

**Figure 11:**
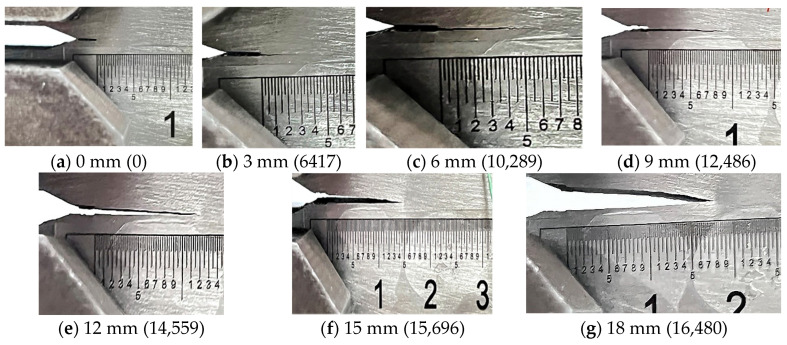
Crack extension process of specimen T3.

During the crack extension of the CT specimen, as the crack length increases, and the distance between PZT1 and PZT2 increases, as do the width and angle of the crack. The shape evolution of the crack in the T3 specimen from 0 mm to 18 mm is illustrated in Figure 11, with the numbers in parentheses indicating the corresponding number of cyclic loadings for the crack.

**Figure 12:**
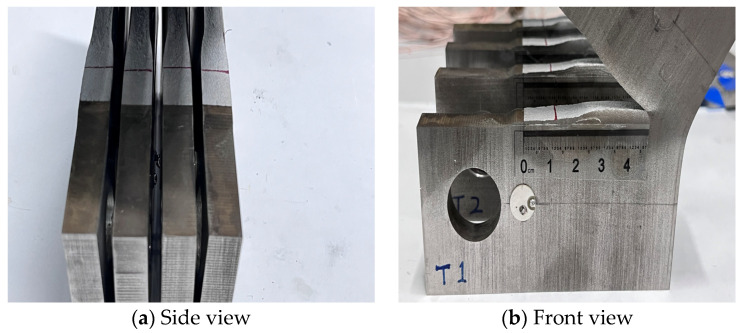
Cracked surface form.

From a stress analysis perspective, the crack is a Type I crack. However, based on the observed angles and widths of the crack openings, the cracks can be further classified as closed cracks (0~9 mm), partially open cracks (12 mm), or fully open cracks (15 mm and 18 mm). Complex wave propagation and boundary reflections can contaminate the received signals, while uncertainties in the local damage geometry shape can affect the received signals [17]. Thus, the open/closed status of the extended crack influences the transmission and scattering of Lamb waves at the crack tip. Between 0 mm and 9 mm, Lamb waves primarily transmit through the crack tip, with some reflection and transmission at 12 mm and only reflection at 15 mm and 18 mm. This behavior results in a monotonic change in the relevant index up to 12 mm, with the monotonicity becoming uncertain after 12 mm. It is observed that a plastic deformation zone is formed around the crack tip during the crack propagation process. The thickness at the tip is thinner than that before 12 mm, leading to complex Lamb wave reflections at this location. As shown in Figure 12, the crack depth of the four specimens generally exhibits necking at approximately 12 mm with rough crack surfaces, causing a change in the monotonicity of the Lamb wave amplitude damage index at 12 mm.

### 3.6. Trends of Each Index and (a-N) Crack Propagation

**Figure 13:**
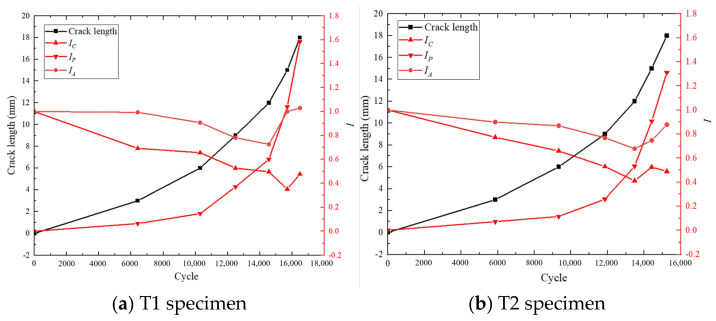

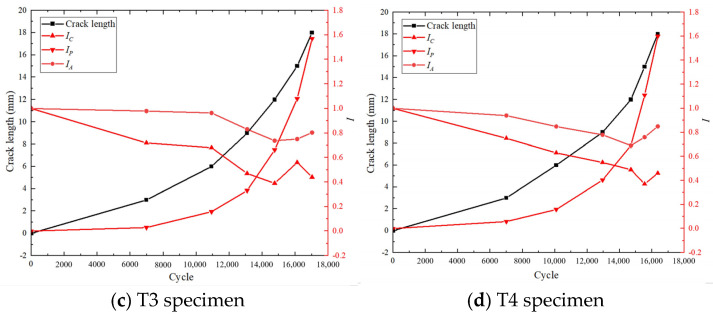
Trends of each index with (*a*-*N*) crack extension.

Based on the calculated indices and the cycle corresponding to cracks of different lengths in the specimens, with the cycle as the horizontal axis, the crack length on the left vertical axis, and the values of various indices on the right vertical axis, the change trends of the indices in the four specimens are similar, as shown in Figure 13. And *I* means the Damage monitoring index.

The number of cyclic loads at 12 mm, 18 mm, and at destruction and the life percentage of our specimens are shown in Table 5. It was found that the number of cycles accounts for more than 80% of the total life when the crack extends to 12 mm. When examining the trend of the amplitude damage index, we found that the crack extension begins to enter the later stage when the index shows an inflection point and the monotonicity begins to change. The number of cycles accounts for more than 95% of the total life when the crack extends to 18 mm, which indicates imminent failure.

### 3.7. Monitoring of Crack Extension Rates by Phase Damage Indices

Figure 13 shows a strong correlation between the curves of crack length versus the number of cycles and the curve of phase damage index versus the number of cycles. By taking the stress intensity factor, Δ*K,* as a conversion parameter, a monitoring relationship for the phase damage index, *I*_p_, and crack growth rate, da/dN, can be established as follows [29]:

(8) $$I_{P}=c_{1}+c_{2}\left(\frac{e^{c_{3}\left(\Delta K\right)}-1}{c_{3}}\right)$$

$I_{p}$ is the phase damage index, which is the dependent variable that needs to be fitted, and ΔK is the stress intensity factor and the independent variable. $c_{1}$ is a constant term representing the phase damage index when ΔK is 0. $c_{2}$ is the amplitude parameter that controls the overall variation amplitude of the phase damage index, and $c_{3}$ is an exponential parameter that controls the rate at which the phase damage index changes with ΔK.

For nonlinear fitting, the stress intensity factor, Δ*K*, that corresponds to crack lengths ranging from 0 mm to 18 mm can be determined from Equation (6), which is used as the horizontal axis. The phase damage index, *I_p_*, is used as the vertical axis. The fitting model is shown in Equation (8), where *c*_1_, *c*_2_, and *c*_3_ are the fitting parameters. The fitting curve is shown in Figure 13, and the parameter values are shown in Table 6. The coefficient of determination (COD) is a coefficient that can be used to measure the fitting effect. The closer the value of COD is to 1, the better the fitting effect.

This fitted curve leads to the relationship between *I_P_* and the stress intensity factor Δ*K* for the four specimens:

(9) $$I_{P}=-0.4605+0.00333\times \left(\frac{e^{0.03715\left(\Delta K\right)}-1}{0.03715}\right)$$

Converting the equation to express Δ*K* as a function of *I_P_* yields the following:

(10) $$\Delta K=\frac{\ln\left[\left(I_{P}+0.4605\right)\times \frac{0.03715}{0.00333}+1\right]}{0.03715}$$

Based on Table 4, the Paris model equation can be expressed as follows:

(11) $$\frac{da}{dN}=2.5679\times 10^{-14}{\left(\Delta K\right)}^{4.2772}$$

**Figure 14:**
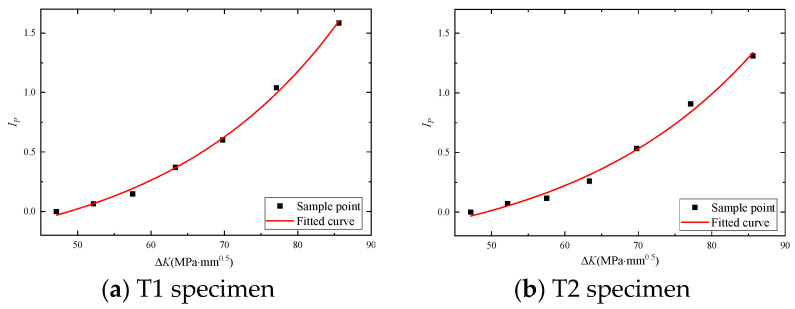

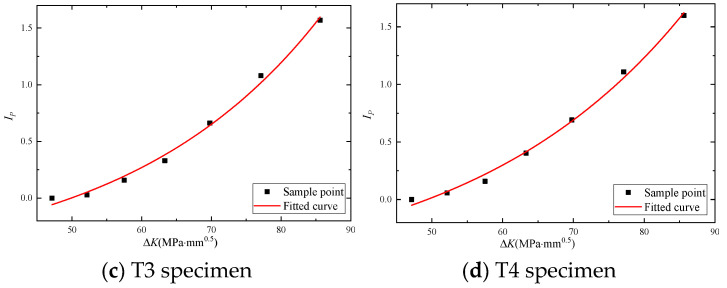
The relationship fitting between Δ*K* and *I_P_*.

Therefore, by calculating the phase damage index of the specimen from the received Lamb wave signal, the stress intensity factor can be determined using Equation (13), which can then be substituted into Equation (13) to obtain the corresponding crack growth rate for the phase damage index. Through calculations, a strong linear relationship between the two is observed, which is expressed as the fitted model given in Equation (12), where *h*_1_ and *h*_2_ are fitting parameters. The fitting parameters and linear regression coefficients are presented in Table 7, and the fitted line is illustrated in Figure 14.

(12) $$\frac{da}{dN}=h_{1}I_{P}+h_{2}$$

**Figure 15:**
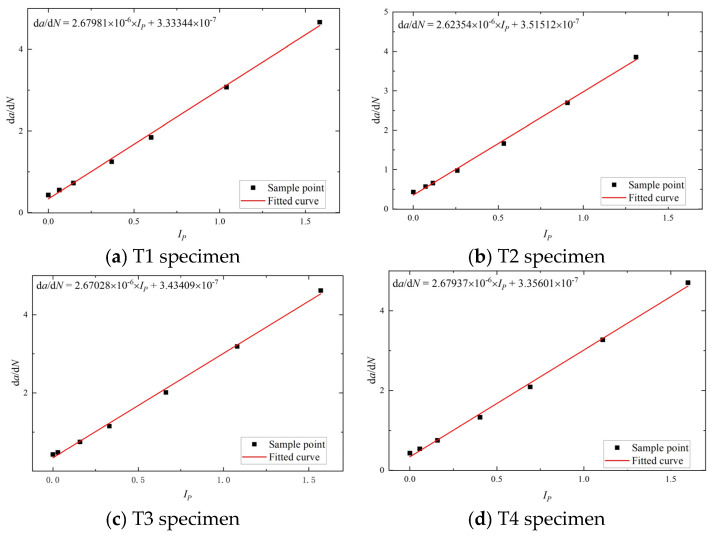
The relationship fitting between *I_P_* and da/dN.

Figure 14 clearly shows that all four specimens exhibit a similar trend with increasing spacing between each data point, mirroring the behavior observed during fatigue crack propagation. Figure 15 shows that when the crack extends to 12 mm, the phase damage index for all four specimens ranges between 0.5 and 0.7. Substituting this relationship into Equation (12) yields crack growth rates ranging from 1.67 × 10^−6^ mm/cycle to 2.21 × 10^−6^ mm/cycle. By utilizing Equation (6), the stress intensity factor can be determined when the crack extends to 12 mm, and further computations following Equation (11) provide a corresponding crack growth rate of 1.96 × 10^−6^ mm/cycle. This rate falls within the range obtained from Equation (13), indicating the model’s prediction accuracy.

Therefore, Equation (12) can be written as follows:

(13) $$\frac{da}{dN}=2.66325\times 10^{-6}\times I_{P}+3.40967\times 10^{-7}$$

## 4. Finite Element Analysis

### 4.1. Model and Preprocessing

The length and width of the finite element plate model are both 600 mm, with a thickness of 10 mm. The material used is 316L steel, and the specific material parameters are shown in Table 1. Set the origin of the coordinate axis at the center of the plate, and arrange PZTs in a 3 × 3 pattern with a horizontal and vertical spacing of 80 mm between two adjacent PZTs. A PZT is a circle with a diameter of 10 mm, and the coordinates of the circular hole damage are set to (120, 0). The coordinates of the actuator are (29 mm, 26 mm) and those for the receiver are (29 mm, 70 mm). Another PZT is symmetrically arranged on the back as the corresponding excitation point of the actuator, while other PZT devices are used as the receiving end. The visualization image of the model and the specific layout of PZTs are shown in Figure 6. This layout helps to capture in detail the signal propagation and reflection caused by circular hole damage for further analysis and evaluation. The excitation signal adopts 2.5 periods, with an excitation frequency of 90 kHz and an antisymmetric *A*_0_ excitation mode. The grid division adopts hexahedral elements (C3D8R) with a grid size of 1 mm and a time increment step set to 100 ns. This layout helps to capture in detail the signal propagation and reflection caused by circular hole damage for further analysis and evaluation.

**Figure 16:**
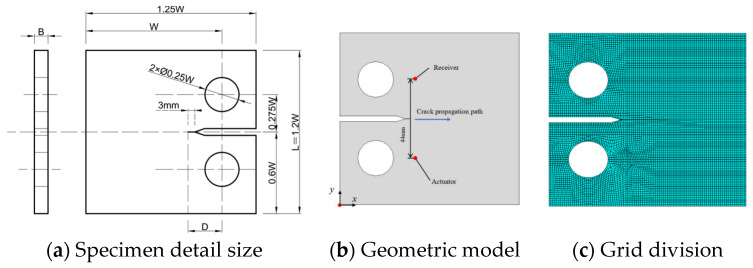
CT specimen design dimensions and finite element modeling and grid division.

The finite element analysis model without cracks, shown in Figure 16, has the following dimensions: W = 80 mm and B = 10 mm. A 3 mm artificial crack, which matches the actual specimen, is positioned at the notch’s top as the crack length is effectively 0 mm, which represents a healthy state where the CT specimen’s crack has not initiated and the specimen remains intact. In this healthy CT specimen, Lamb waves propagate along the crack propagation path without penetrating it. Upon crack formation, the Lamb waves interact with the crack, producing damage scattering signals. To investigate the variations in Lamb wave crack transmission and reflection signals during the fatigue crack propagation of the CT specimen, which allows for a clearer observation of the influence of crack length on Lamb wave signals, PZT1 and PZT2 are arranged on both sides in the crack propagation direction, with specific placement details as shown in the Figure 16. PZT1 serves as the excitation end using dual-sided excitation, while PZT2 acts as the receiving end. In the absence of a crack, the direct distance between PZT1 and PZT2 is 44 mm.

As the crack in the CT specimen extends, the model is also adjusted accordingly. The model in this study is based on the dimensions of the actual experiments, primarily changing the crack width between two circular holes recorded by the fatigue testing machine. The modeled crack size ranges from 0 mm to 18 mm, with intervals of 3 mm. The excitation signal was subjected to the same 2.5 periods as in the experiment, with an excitation frequency of 90 kHz and using the antisymmetric *A*_0_ excitation mode.

**Figure 17:**
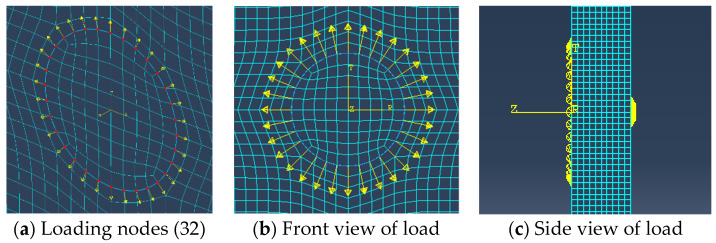
PZT excitation load arrangement in the simulation.

Figure 16c shows the simplification of the excitation process of the signal in the simulation. This simplification can reduce the complexity of the simulation while ensuring sufficient accuracy and efficiency. In Figure 17, a concentrated load is applied circumferentially in the tangential direction of the steel plate, with the loading point located at the outer diameter node of the PZT. Thirty-two concentrated loads are arranged, with the load varying over time and following a sine wave excitation pattern.

### 4.2. Mesh Size Calculation

Meshing is required at the piezoelectric ceramic piece to achieve effective excitation of Lamb waves. The plate is both transversely and longitudinally meshed to ensure uniform meshing. The size of the mesh division is determined by the minimum wavelength, calculated specifically as follows:

Transverse wave velocity:

(14) $$c_{T}=\sqrt{\frac{\mu }{\rho }}=\sqrt{\frac{E}{2\rho \left(1+\nu \right)}}=\sqrt{\frac{2.16\times 10^{11}\mathrm{Pa}}{2\times 7980\times \left(1+0.3\right){\mathrm{kg}/m}^{3}}}=3226.55m/s$$

Transverse wave wavelength:

(15) $$\lambda_{T}=\frac{c_{T}}{f}=\frac{3248.88m/s}{90\mathrm{kHz}}=35.85\mathrm{mm}$$

The minimum wavelength should be ensured through 10 units:

(16) $$l\leq \frac{\lambda_{\min}}{10}$$

where *l* represents the grid size, and $\lambda_{\min}$ represents the minimum wavelength. Therefore,

(17) $$l\leq \frac{\lambda_{\min}}{10}=\frac{\min\left(\lambda_{L},\lambda_{T}\right)}{10}=\frac{35.85}{10}=3.585\mathrm{mm}$$

The thickness of the plate is 10 mm. To achieve accurate and uniform division, grid sizes in this study are selected as 1 mm.

### 4.3. Analysis of Lamb Wave Propagation Process

**Figure 18:**
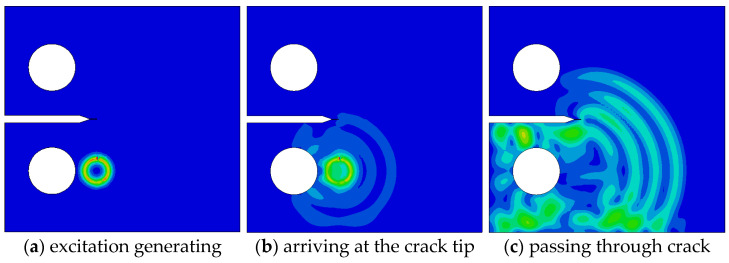
Lamb wave propagation for a fatigue crack of 0 mm.

**Figure 19:**
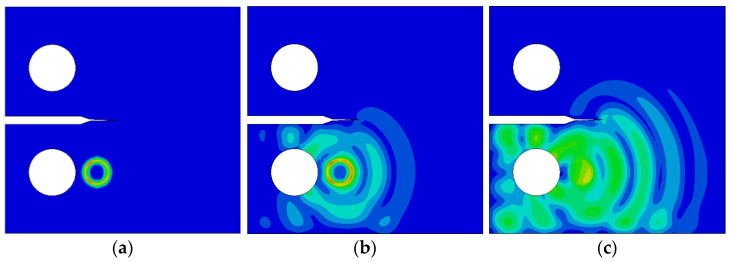
Lamb wave propagation for a fatigue crack of 9 mm: (**a**) excitation generating, (**b**) arriving at the crack tip, and (**c**) passing through the crack.

**Figure 20:**
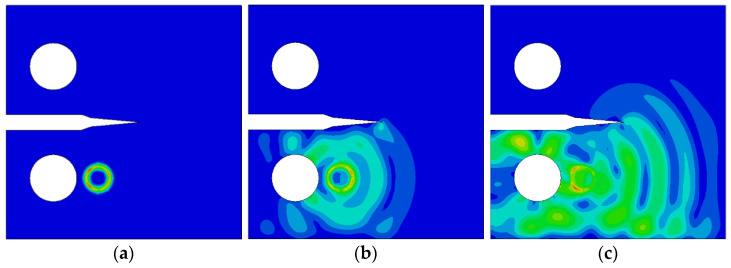
Lamb wave propagation for a fatigue crack of 18 mm: (**a**) excitation generating, (**b**) arriving at the crack tip, and (**c**) passing through the crack.

The crack growth simulates Lamb wave propagation for seven crack lengths ranging from 0 mm to 18 mm. The propagation process includes the excitation signal at the crack tip, crack tip propagation, and passage through the crack, as shown in Figure 18, Figure 19 and Figure 20.

If the excitation center is connected to the crack tip set at the notch tip in a straight line, then the line is parallel to the left and right boundaries of the specimen when the crack is not developed; thus, in Figure 18b, Lamb waves arrive directly at the crack tip without any transmission or reflection phenomena. As shown in Figure 18c, the stress waves in the outer ring are relatively uniform. When the crack is 9 mm, as shown in Figure 19b, Lamb waves encounter reflection at the boundary at one end of the specimen’s notch where the crack is located when they propagate to the crack. Transmission and reflection phenomena exist at the crack tip, which delay the direct wave signal. 

As shown in Figure 19c, the stress waves in the outer ring are not uniform, which proves that the Lamb wave signals propagating to the right are faster than those propagating to the left. When the crack width is 18 mm, the crack width is maximal, and the angle at the crack tip is also the largest among the seven simulated cases. As shown in Figure 20b, when Lamb waves arrive at the crack tip, the reflection phenomena are more pronounced than before, and transmission phenomena are almost absent, leading to more of a time delay. The variation pattern of stress waves is consistent with the patterns observed in Lamb wave damage identification simulations; when the excitation signal is triggered, the stress wave is red, turning green during propagation, and appearing light blue after encountering damage, with the stress waves in the outer ring also turning light blue. Therefore, energy is lost during the propagation of Lamb waves and upon encountering a crack.

**Figure 21:**
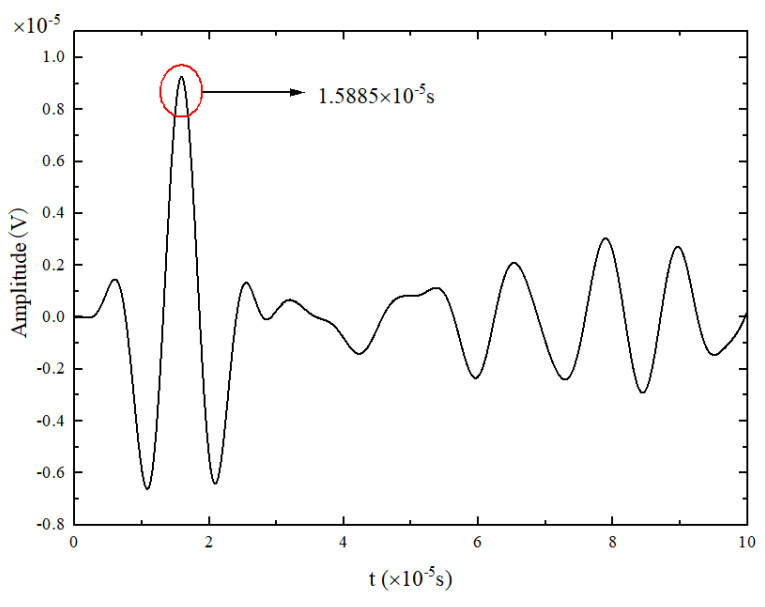
Excitation signal at PZT1.

**Figure 22:**
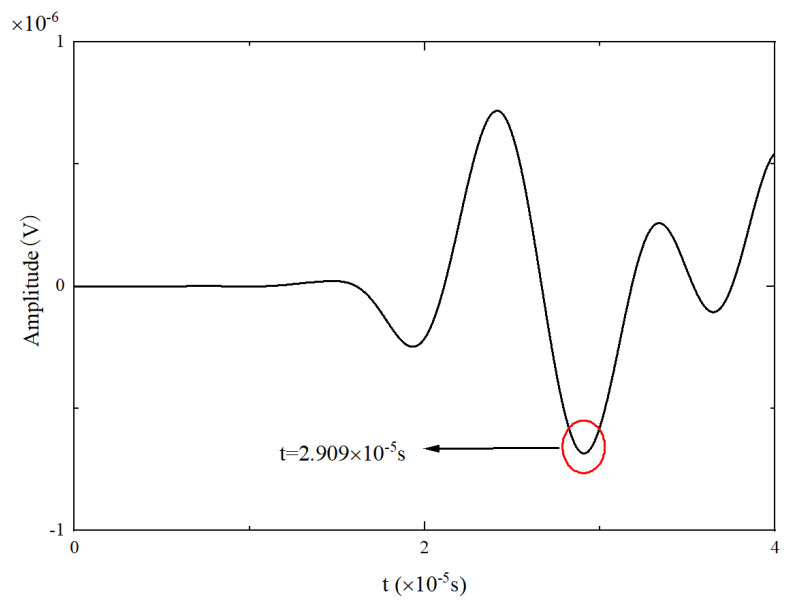
Received signal at PZT2.

The received signal at 0 mm was analyzed to verify the accuracy of the simulation, and the time of the maximum wave peak was taken as the arrival time of the wave packet. The excitation method used in this numerical simulation is antisymmetric excitation, i.e., the A_0_ mode. As shown in Figure 4, when the frequency is 90 kHz, the group velocity of the A_0_ mode Lamb wave is 3211.54 m/s. Figure 21 shows the excitation signal at PZT1, with the time of the center peak of the first wave as the departure time of the wave packet, i.e., $1.5885\times 10^{-5}s$. Figure 22 shows the Lamb wave signal received at PZT2, where the first wave packet is the direct wave, arriving at $2.909\times 10^{-5}s$. Therefore, the flight time of Lamb wave propagation through damage is the difference between these two times, i.e., $\Delta t=1.3205\times 10^{-5}s$. The straight-line distance between PZT1 and PZT2 is 44 mm; hence, the calculated group velocity of the simulation is as follows:

(18) $$v=\frac{l}{\Delta t}=\frac{44\mathrm{mm}}{1.3205\times 10^{-5}s}=3332.07m/s$$

A comparison with the theoretical group velocity of 3211.54 m/s shows an error of 3.75%, which is within an acceptable range and indicates that the numerical simulation is reasonable. A trend analysis of various crack signal features will be carried out next.

### 4.4. Change in the Damage Index of Lamb Waves

**Figure 23:**
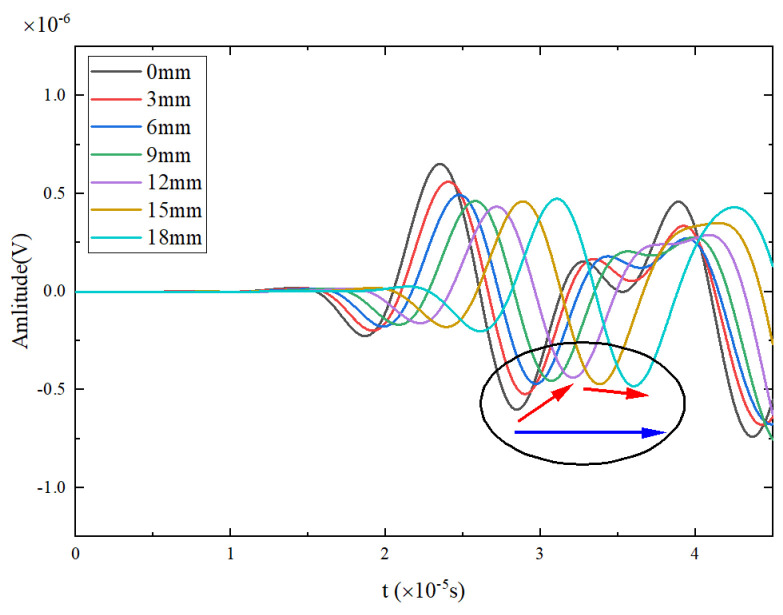
Monitoring signals for PZT1-PZT2 obtained in simulation.

The crack length gradually increases with the crack width, while the peak time of the direct wave signal is gradually delayed (blue arrow in Figure 23), which indicates a phase shift, a change in the phase damage index. The variation in the amplitude of the direct wave does not show a monotonic pattern; it first decreases and then increases, reaching its minimum when the crack extends to 12 mm (red arrow in Figure 23).

**Figure 24:**
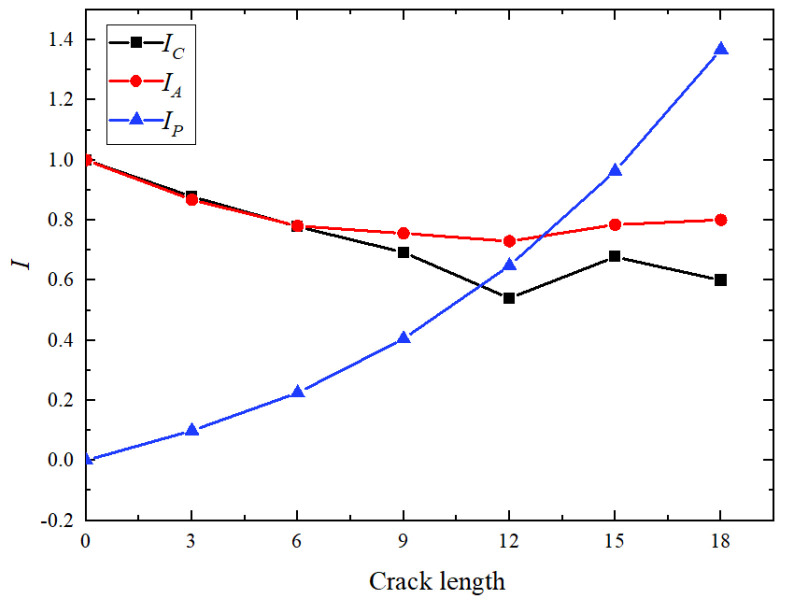
Variation in each index with crack length obtained in simulation.

The Lamb wave signal correlation coefficient and damage index at the receiver end are calculated according to Equations (1), (2) and (3), respectively. The relationship between each value and the crack length is shown in Figure 24.

The black curve in Figure 24 demonstrates that the correlation coefficient decreases with crack extension, but the relationship is not strictly monotonic. During the extension from 0 mm to 12 mm, the correlation coefficient decreases with crack growth and almost shows a linear relationship. Above 12 mm, the change in the correlation coefficient is related to crack extension, indicating that variations in the crack angle and width influence the propagation of Lamb waves at the crack tip. The red curve in Figure 24 shows that the amplitude damage index first decreases and then increases with crack length, reaching a minimum when the crack extends to 12 mm. The change in amplitude is very noticeable as the crack extends from 0 mm to 3 mm, followed by a gradual decrease in the rate of change. The blue curve in Figure 24 reveals that the phase damage index increases monotonically with crack length, with a relatively gentle change from 0 mm to 9 mm and a faster rate of change after 9 mm.

## 5. Conclusions

This paper as explored the crack propagation in CT specimens based on Lamb waves, combining experimental and finite element simulations to derive the relevant laws of Lamb wave propagation at different crack lengths. The specific conclusions are as follows:

(1) The variation in the damage index is highly correlated with changes in the crack. When the crack width and angle are small, Lamb waves predominantly propagate at the crack tip through transmission and reflection. As the crack enlarges, Lamb waves mainly propagate through reflections at the crack tip. The propagation form at the crack tip changes by approximately 12 mm.
(2) (2) The numerical simulations employed simplifications. Notably, the transition of the model from an intact to a cracked state was not simulated. Instead, models with crack sizes of 0 mm, 3 mm, and 6 mm were directly established based on experimental measurements of the specimen dimensions. Therefore, the numerical simulation results exhibit a linear trend and do not show a particularly sensitive pattern.
(3) A novel conclusion drawn from this experiment is that fatigue already exceeds 80% of the total life when the amplitude damage index reaches a minimum value (at 12 mm crack extension). Constructing a crack propagation rate monitoring model based on the phase damage index revealed a strong linear relationship between the phase damage index and crack propagation rate, which validates the feasibility of using Lamb waves to monitor the crack propagation rate. This finding provides a reference for monitoring methods of fatigue crack propagation in actual engineering for bridges.

Further research is needed on the following issues:

(1) The propagation of Lamb waves at the crack tip needs further research, and it is necessary to quantitatively study the crack width and crack growth angle when the propagation of Lamb waves changes.
(2) The change in Lamb wave signal damage index reflects fatigue life, and further exploration should include visualizing the damage index more concisely.

## Figures and Tables

**Table 1:** Parameters of the 316L steel plate material.

Poisson’s Ratio ν	Young’s Modulus E(GPa)	Longitudinal Wave Velocity $c_{L}\left(m/s\right)$	Transverse Wave Velocity $c_{T}\left(m/s\right)$	Density $\rho \left(\mathrm{kg}/m^{3}\right)$
0.3	216.43	6036.33	3226.55	7980

**Table 2:** Load and dimensions of CT specimens.

Specimen Number	Stress Ratio	Thickness B (mm)	Width W (mm)	Crack Length a (mm)	Payload Range ∆F (kN)
T1	0.1	10.04	80.0	19.02	27
T2	0.1	10.02	80.0	19.05	27
T3	0.1	10.06	80.0	19.06	27
T4	0.1	10.04	80.0	19.03	27

**Table 3:** Parameters of each specimen obtained from the fitting.

Specimen Number	Stress Ratio	lg*C*	*m*	Correlation Coefficient	Paris Formula
T1	0.1	−13.6073	4.2974	0.9978	da/dN = 2.4700 × 10^−14^Δ*K*^4.2974^
T2	0.1	−13.5233	4.2414	0.9989	da/dN = 2.9971 × 10^−14^Δ*K*^4.2414^
T3	0.1	−13.7270	4.3334	0.9957	da/dN = 1.8750 × 10^−14^Δ*K*^4.3334^
T4	0.1	−13.5332	4.2365	0.9973	da/dN = 2.9295 × 10^−14^Δ*K*^4.2365^

**Table 4:** The mean and variance of each parameter obtained from the fitting.

	lg*C*	*m*	Correlation Coefficient	Paris Formula
Average	−13.5977	4.2772	0.9974	da/dN = 2.5679 × 10^−14^ΔK^4.2772^
Variance	0.0942	0.04662	0.00132	

**Table 5:** Number of cycles and their percentages.

Specimen Number	Cycle at 12 mm	Cycle at 18 mm	Cycle at Destruction	Percentage of Cycles at 12 mm	Percentage of Cycles at 18 mm
T1	14,559	16,480	16,931	85.99%	97.34%
T2	13,481	15,239	15,635	86.22%	97.47%
T3	14,795	17,027	17,767	83.27%	95.83%
T4	14,701	16,366	16,947	86.75%	96.57%

**Table 6:** Fitting parameters and COD.

	*c*_1_	*c*_2_	*c*_3_	COD
T1	−0.38942	0.00246	0.04134	0.99717
T2	−0.36055	0.0024	0.03922	0.99168
T3	−0.51555	0.00374	0.03563	0.9939
T4	−0.57649	0.00473	0.03244	0.9954
Average value	−0.46050	0.00333	0.03715	0.99454
Sample variance	0.10252	0.00112	0.00392	0.002332

**Table 7:** Fitting parameters and linear regression coefficients.

	*h*_1_	*h*_2_	Linear Regression Coefficient
T1	2.67981 × 10^−6^	3.33344 × 10^−7^	0.99877
T2	2.62354 × 10^−6^	3.51512 × 10^−7^	0.99885
T3	2.67028 × 10^−6^	3.43409 × 10^−7^	0.9988
T4	2.67937 × 10^−6^	3.35601 × 10^−7^	0.99881
Average value	2.66325 × 10^−6^	3.40967 × 10^−7^	0.99881
Sample variance	(2.68353 × 10^−8^)^2^	(8.24751 × 10^−9^)^2^	(3.30404 × 10^−5^)^2^

## Data Availability

The original contributions presented in the study are included in the article, further inquiries can be directed to the corresponding authors.
